# Wnt signalling in human breast cancer: expression of the putative Wnt inhibitor Dickkopf-3 (*DKK3*) is frequently suppressed by promoter hypermethylation in mammary tumours

**DOI:** 10.1186/bcr2151

**Published:** 2008-09-30

**Authors:** Jürgen Veeck, Nuran Bektas, Arndt Hartmann, Glen Kristiansen, Uwe Heindrichs, Ruth Knüchel, Edgar Dahl

**Affiliations:** 1 Molecular Oncology Group, Institute of Pathology, University Hospital of the RWTH, Aachen, Pauwelsstrasse 30, D-52074 Aachen, Germany; 2 Department of Pathology, University of Erlangen, Krankenhausstrasse 12, D-91054 Erlangen, Germany; 3 Institute of Surgical Pathology, University Hospital Zürich, Schmelzbergstrasse 12, CH-8091 Zürich, Switzerland; 4 Department of Gynecology, Breast Surgery and Mastology, University Hospital of the RWTH Aachen, Pauwelsstrasse 30, D-52074 Aachen, Germany; 5 Brustzentrum Aachen-Stadt, Pauwelsstrasse 30, D-52074 Aachen, Germany

## Abstract

**Introduction:**

Expression of the putative Wnt signalling inhibitor Dickkopf-3 (*DKK3*) is frequently lost in human cancer tissues because of aberrant 5'-cytosine methylation within the *DKK3 *gene promoter. Since other Wnt signalling inhibitors have been reported to be targets of epigenetic inactivation in human breast cancer, we questioned if *DKK3 *expression is also epigenetically silenced during breast carcinogenesis and therefore might contribute to oncogenic Wnt signalling commonly found in this disease.

**Methods:**

*DKK3 *mRNA expression and *DKK3 *promoter methylation were determined by RT-PCR, realtime PCR and methylation-specific PCR in breast cell lines (n = 9), normal breast tissues (n = 19) and primary breast carcinomas (n = 150), respectively. *In vitro *DNA demethylation was performed by incubating breast cell lines with 5-aza-2'-deoxycytidine and trichostatin A. DKK3 protein expression was analysed by immunohistochemistry in breast carcinomas (n = 16) and normal breast tissues (n = 8). Methylation data were statistically correlated with clinical patient characteristics. All statistical evaluations were performed with SPSS 14.0 software.

**Results:**

*DKK3 *mRNA was downregulated in 71% (five of seven) of breast cancer cell lines and in 68% of primary breast carcinomas (27 of 40) compared with benign cell lines and normal breast tissues, respectively. A DNA demethylating treatment of breast cell lines resulted in strong induction of *DKK3 *mRNA expression. In tumourous breast tissues, *DKK3 *mRNA downregulation was significantly associated with *DKK3 *promoter methylation (p < 0.001). Of the breast carcinomas, 61% (92 of 150) revealed a methylated *DKK3 *promoter, whereas 39% (58 of 150) retained an unmethylated promoter. Loss of DKK3 expression in association with *DKK3 *promoter methylation (p = 0.001) was also confirmed at the protein level (p < 0.001). In bivariate analysis, *DKK3 *promoter methylation was not associated with investigated clinicopathological parameters except patient age (p = 0.007).

**Conclusions:**

*DKK3 *mRNA expression and consequently DKK3 protein expression become frequently downregulated during human breast cancer development due to aberrant methylation of the *DKK3 *promoter. Since DKK3 is thought to negatively regulate oncogenic Wnt signalling, *DKK3 *may be a potential tumour suppressor gene in normal breast tissue.

## Introduction

The mammalian Dickkopf genes (*DKK*) encode a class of extracellular signalling molecules that control cell fate during embryonic development and regulate tissue homeostasis in adults [1,2]. Four DKK gene members have been identified so far. *DKK1*, *DKK2 *and *DKK4 *antagonise canonical Wnt/β-catenin signalling by interaction with LDL-receptor-related proteins (LRP5 and LRP6) [3]. In contrast, *DKK3 *does not sequester LRPs or Wnt ligands [2,4,5]. Its function in antagonising nuclear β-catenin levels, designated as the hallmark of an activated Wnt pathway often found in human tumour tissues [6], has received conflicting reports [7-9]. Most evidence suggest *DKK3 *exerts a tumour suppressive function by inhibiting a non-canonical Wnt signalling branch referred to as the planar cell polarity (PCP) pathway. The PCP pathway is characterised by the activation of c-Jun kinase (JNK) via recruitment of small GTPases of the Rho/Rac family [10]. It results in changes in cell adhesion, motility and polarity [11] rather than interfering with the networks of proliferation and differentiation, which is mediated by canonical Wnt/β-catenin signalling [6].

In agreement with its putative tumour-suppressive function [9,12-14]*DKK3 *is commonly downregulated in human cancers such as lung cancer [15-17], renal clear cell carcinoma [18], pancreatic cancer [19], leukaemia [20], prostate cancer [7,21], bladder cancer [22], melanoma [23] and gastrointestinal tumours [24]. In many of these diseases transcriptional loss is tightly associated with methylation of the *DKK3 *promoter [15,16,18,20-22,24], whereas in other malignancies the cause of downregulation remains to be elucidated or is not related to 5'-cytosine methylation [23]. A study on lung cancer revealed that the rate of *DKK3 *methylation increased steadily from normal lung tissue, to low-grade and high-grade atypical adenomatous hyperplasia to invasive adenocarcinoma [25], suggesting a potential role of *DKK3 *methylation in lung cancer progression. In mouse cancer models, *DKK3 *has proved a promising therapeutic agent capable of repressing tumour progression, for example, in testicular germ cell cancer [14] and prostate cancer [13]. More recently, a breast cancer xenotransplantation model demonstrated that a single adenoviral-mediated intra-tumoural injection of a *DKK3 *expression vector efficiently discontinued tumour growth, with the induction of apoptosis in these cells [26]. This suggests that *DKK3 *may have an important tumour-suppressive function that either prevents tumour initiation or attenuates cancer progression. Interestingly, loss of *DKK3 *expression was first observed in numerous immortalised tumour-derived cell lines [27]. Immortalisation, that is escape from cellular senescence, is an early event in malignant transformation [28], so *DKK3 *could act as a tumour suppressor gene by mediating the effects of senescence stimuli. In concordance with this hypothesis, *DKK3 *expression was found to be elevated in organs with predominantly growth-arrested post-mitotic cells, for example in the heart and brain [29] and also in senescent prostate epithelial cells [30].

However, to the authors' knowledge, a comprehensive study on *DKK3 *gene regulation and its implication in breast cancer has not yet been published. In our study we investigated *DKK3 *mRNA expression, DKK3 protein expression and *DKK3 *promoter methylation in breast cell lines as well as in normal and malignant primary breast tissues. Our results demonstrate for the first time that *DKK3 *expression is frequently downregulated in human breast cancer as a consequence of aberrant DNA methylation within the *DKK3 *gene promoter.

## Materials and methods

### Patient material

Nineteen matched tumour and normal tissue samples from breast cancer patients and 131 tissue samples from breast carcinomas were obtained from patients treated by primary surgery for breast cancer at the Departments of Gynecology at the University Hospitals of Aachen, Jena, Regensburg and Düsseldorf, Germany, between 1991 and 2005. The selection of cases was based on availability of tissue, and the sample was recruited in a non-selective, consecutive manner. Female patients presenting with unilateral, invasive breast cancer with no individual breast cancer history were included in the study. Exclusion criteria were: neo-adjuvant chemotherapy before surgery; presentation with secondary breast cancer; or peritumourous carcinoma *in situ *present in the tumour sample. Patient characteristics are shown in Table 1.

**Table 1:** Demographic and clinical patient characteristics of primary breast carcinomas (n = 150)

**Variable**	**Categorisation**	**n**^1^	**%**
***Clinicopathological factors***			
Age at diagnosis			
	median: 57 years (range 28 to 86 years)		
	<57 years	74	49.3
	= 57 years	76	50.7
Tumour size^2^			
	pT1	59	39.3
	pT2	70	46.7
	pT3	8	5.3
	pT4	10	6.7
	pTx	3	2.0
Lymph node status^2^			
	pN0	72	48.0
	pN1 to 3	71	47.3
	PNx	7	4.7
Histological grade			
	G1	13	8.7
	G2	75	50.0
	G3	62	41.3
Histological type			
	Ductal	122	81.3
	Lobular	19	12.7
	Other	9	6.0
			
***Immunohistochemistry***			
Oestrogen receptor			
	Negative (IRS^3 ^0 to -2)	47	31.3
	Positive (IRS 3 to 12)	98	65.3
	n.a.	5	3.3
Progesterone receptor			
	Negative (IRS^3 ^0 to 2)	51	34.0
	Positive (IRS 3 to 12)	94	62.7
	n.a.	5	3.3

^1^Only female patients with unilateral invasive breast cancer were included. ^2^According to International Union Against Cancer: TNM Classification of Malignant Tumours [32].^3^IRS = immunoreactivity score [35]. n.a. = not available.

All patients gave informed consent for retention and analysis of their tissue for research purposes and the Institutional Review Boards of the participating centres approved the study.

Tumour histology was determined according to the criteria of the World Health Organization [31], while disease stage was assessed according to International Union Against Cancer [32]. Tumours were graded according to Bloom and Richardson, as modified by Elston and Ellis [33]. After surgery, tumour material was immediately snap-frozen in liquid nitrogen. Sections stained with haematoxylin and eosin were prepared for assessing the percentage of tumour cells; only samples containing more than 70% tumour cells were selected. Samples were dissolved in lysis buffer followed by DNA isolation using the QIAamp DNA Mini Kit (Qiagen, Hilden, Germany) according to the manufacturer's recommendations.

### Breast cell lines

The cancerous breast cell lines BT20, Hs578T, MCF7, MDA-MB231, SKBR3, T47D and ZR75-1 and the non-cancerous lines HMEC and MCF12A were obtained from the American Type Culture Collection (ATCC) (Rockville, MA, USA) and cultured under recommended conditions.

### RNA expression analysis

RNA isolation, RT-PCR and realtime PCR were performed as previously described [34]. To ensure experiment accuracy, all quantitative measurements were performed in triplicate. Intron-spanning primer sequences and cycling conditions are given in Table 2.

**Table 2:** Oligonucleotide primers used in the study

	**Sequence (5' to 3')**	**T_A _[°C]**	**Primer [nM]**	**Cycles**	**Product size (bp)**
***RT-PCR***					

GAPDH	Forward: TGGTCACCAGGGCTGCTT				
		59	400	35	510
	Reverse: GTCTTCTGGGTGGCAGTGAT				

					

DKK3	Forward: AAGGCAGAAGGAGCCACGAGTGC				
		59	400	35	182
	Reverse: GGCCATTTTGGTGCAGTGACCCCA				

***Semiquantitative realtime PCR***					

GAPDH	Forward: ATGGCCAGCGGGAAGAC				
		60	500	40	289
	Reverse: ATGGCCAGCGGGAAGAC				

					

DKK3	Forward: ACAGCCACAGCCTGGTGTA				
		60	400	40	123
	Reverse: CCTCCATGAAGCTGCCAAC				

***Methylation-specific PCR***					

DKK3 unmethylated	Forward: TTAGGGGTGGGTGGTGGGGT				
		58	320	34	126
	Reverse: CTACATCTCCACTCTACACCCA				

					

DKK3 methylated	Forward: GGGCGGGCGGCGGGGC				
		58	320	34	120
	Reverse: ACATCTCCGCTCTACGCCCG				

T_A _= annealing temperature.

### Bisulphite-modification and methylation-specific PCR

About 1 μg of genomic DNA was bisulphite-modified using the EZ DNA Methylation Kit (Zymo Research, Orange, CA, USA) according to the manufacturer's recommendations. Methylation-specific PCR (MSP) was performed as previously described [34]. Primers and cycling conditions are listed in Table 2. Specificity of MSP primers in detecting the *DKK3 *methylation status were demonstrated using universal unmethylated and universal polymethylated DNA as a template (Epi Tect Control DNA Set; Qiagen, Hilden, Germany).

### 5-aza-2'-deoxycytidine and trichostatin A treatment

DNA demethylating treatment of breast cancer cell lines was performed with 5-aza-2'-deoxycytidine as described elsewhere [34], modified by the addition of 300 nM of the histone deacetylase inhibitor trichostatin A (Sigma-Aldrich, Deisenheim, Germany) on day three (incubation for 24 hours). Drug concentrations were adjusted in advance to warrant viability and replication of all cell lines.

### Immunohistochemistry

Formalin-fixed, paraffin-embedded 2 μm tissue sections were subjected to immunostaining using the Advance Kit (Dako, Hamburg, Germany) following the manufacturer's instructions. Antigen retrieval was performed by pre-treatment with citrate buffer (pH 7) in a microwave oven (20 minutes at 200 W). Sections were incubated for 30 minutes with the primary antibody (AP1523a, Abgent, San Diego, CA, USA; 1:50), washed and incubated for 10 minutes with the secondary antibody (biotinylated polylink; Dako, Hamburg, Germany). Diaminobenzidin (Dako, Hamburg, Germany) was used for antibody detection. An experienced breast cancer pathologist (N.B.) scored the immunohistochemical staining according to the scoring system suggested by Remmele and Stegner [35]. The tissue specimens for immunohistochemical analysis are characterised in Table 3.

**Table 3:** Immunohistochemical analysis of DKK3 protein expression in breast cancer samples (n = 16) in relation to clinicopathological parameters

**Age**	**Size^1 ^(pT)**	**Node status^1 ^(pN)**	**Grade**	**Histology**	**ER^2^**	**PR^2^**	***DKK3 *methylation**	**DKK3 IRS tumour^3^**	**DKK3 IRS normal^4^**
49	1	0	3	IDC	-	-	-	9	12
85	2	1	3	IDC	-	-	n.a.	3	n.a.
69	2	1	2	ILC	-	-	-	9	12
82	3	1	3	IDC	-	-	+	6	n.a.
45	1	1	3	IDC	-	-	+	4	12
60	3	0	3	IDC	-	-	n.a.	8	n.a.
61	1	0	2	IDC	-	-	+	0	n.a.
72	1	0	2	IDC	-	-	n.a.	4	n.a.
44	1	1	3	IDC	-	-	-	12	12
86	2	n.a.	3	IDC	-	-	-	8	n.a.
60	1	1	3	IDC	+	-	+	2	9
66	1	0	2	IDC	+	+	+	3	n.a.
54	2	0	3	IDC	+	-	+	6	12
83	1	0	2	IDC	+	+	-	9	n.a.
79	2	0	3	IDC	+	+	-	8	12
62	4	1	3	IDC	+	-	+	3	9

^1^According to International Union Against Cancer: TNM Classification of Malignant Tumours [32]. ^2^According to Remmele and Stegner [35]. ^3^According to Remmele and Stegner [35] as a continuous variable. ^4^Matched normal breast epithelial tissue. IRS = immunoreactivity score. IDC = invasive ductal carcinoma. ILC = invasive lobular carcinoma. n.a. = not available.

### Statistical evaluations

Statistical analyses were completed using SPSS 14.0 (SPSS, Chicago, IL, USA). Differences were considered significant when p < 0.05. A non-parametric Mann-Whitney U-test and a Kruskal-Wallis test were applied to examine expression levels among normal tissues, *DKK3 *unmethylated tumours and *DKK3 *methylated tumours. A student's t-test was applied on the expression of paired normal and tumour samples. To study statistical associations between clinicopathological factors and *DKK3 *methylation status contingency tables and a two-sided Fisher's exact test were used.

## Results

### Differential *DKK3 *mRNA expression in breast cell lines

**Figure 1:**
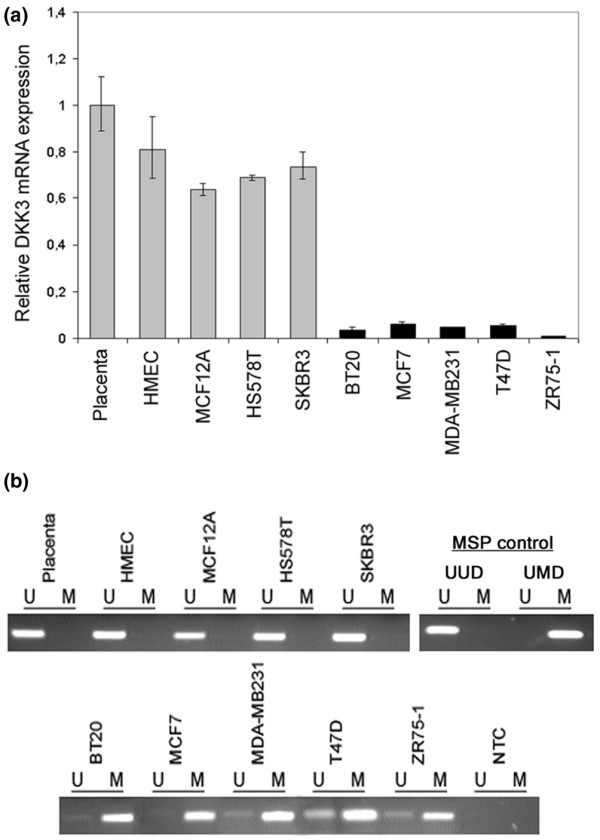
***DKK3 *mRNA expression and *DKK3 *promoter methylation analysis in breast cell lines**. **(a) ***DKK3 *mRNA expression is differentially expressed in breast cell lines. Non-cancerous cell lines (HMEC and MCF12A) and cancerous breast cell lines (Hs578T, SKBR3, BT20, MCF7, MDA-MB231, T47D and ZR75-1) were analysed by realtime PCR and related to *DKK3 *mRNA expression in human placental tissue (set to 1). Grey bars = samples expressing abundant *DKK3 *mRNA; black bars = samples showing strong downregulation of *DKK3 *expression. **(b) **Hypermethylation of the *DKK3 *promoter in breast cell lines. Methylation specific PCR (MSP) was performed with bisulphite-treated DNA from benign and malignant breast cell lines. DNA bands in lanes labelled with U indicate PCR products amplified with primers recognising unmethylated *DKK3 *promoter sequence. DNA bands in lanes labelled with M represent amplificate generated with methylation-specific primers; water served as 'no template control' (NTC). MSP controls demonstrate the specificity of the *DKK3 *primers used. Universal poly-methylated bisulphite-converted DNA (UMD) exclusively yields amplification products with primers specific to methylated *DKK3 *promoter sequence; universal unmethylated bisulphite-converted DNA (UUD) yields exclusive amplification products with primers recognising the unmethylated *DKK3 *promoter sequence.

To start our analysis of *DKK3 *expression in breast cancer patients we analysed mRNA expression in non-malignant and malignant breast cell lines using realtime PCR. Strong *DKK3 *expression could be detected in non-malignant HMEC and MCF12A cells (Figure 1a) exhibiting expression levels comparable with human placental tissue, which is known to abundantly express *DKK3 *[22]. Among the malignant cell lines, Hs578T and SKBR3 cells revealed abundant *DKK3 *mRNA levels comparable with benign breast cells. In five further breast cancer cell lines (BT20, MCF7, MDA-MB231, T47D and ZR75-1) *DKK3 *expression was substantially reduced.

### Methylation of the *DKK3 *promoter in breast cell lines

Aberrant promoter hypermethylation of tumour suppressor genes during carcinogenesis is an effective mechanism resulting in downregulation and functional inactivation of these genes [36,37]. Knowing that *DKK3 *expression was downregulated in most malignant breast cell lines we performed promoter methylation analysis in these cells. By using MSP [38] we found a methylated *DKK3 *promoter sequence in all cell lines showing reduced *DKK3 *expression, BT20, MCF7, MDA-MB231, T47D and ZR75-1 (Figure 1b). In contrast to this, all *DKK3 *expressing cell lines (HMEC, MCF12A, Hs578T and SKBR3) as well as human placental tissue lacked *DKK3 *promoter methylation in the analysed promoter region.

### *In vitro *demethylation of the *DKK3 *promoter

**Figure 2:**
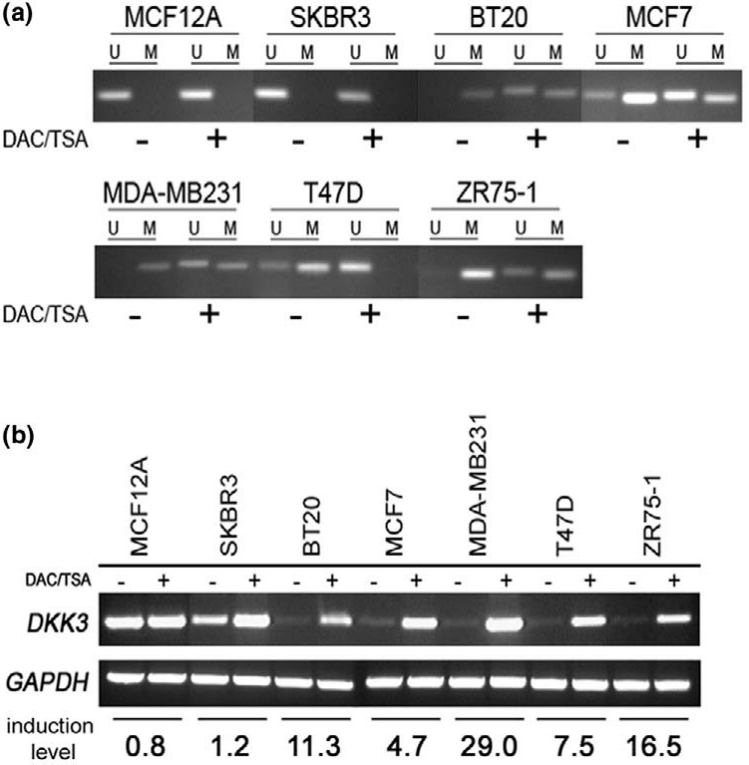
***DKK3 *mRNA expression after *in vitro *DNA demethylation**. **(a) ***In vitro *demethylation of the *DKK3 *promoter. Methylation specific PCR of seven breast cell lines was performed with DNA from cells either untreated (-) or treated with 1 μM 5-aza-2'-deoxycytidine (DAC) and 300 nM trichostatin A (TSA) (+). **(b) **Re-expression of *DKK3 *mRNA in breast cell lines after treatment with DAC/TSA. All cell lines initially exhibiting low *DKK3 *mRNA restored expression of *DKK3 *compared with the untreated control cells. Induction level (fold change) of *DKK3 *mRNA expression was determined by realtime PCR.

To prove a direct association of *DKK3 *promoter methylation with loss of *DKK3 *mRNA expression we treated seven breast cell lines sequentially with the DNA-methyltransferase inhibitor 5-aza-2'-deoxycytidine and the histone deacetylase inhibitor trichostatin A. Subsequently, we determined *DKK3 *promoter methylation and *DKK3 *mRNA expression before and after the drug treatment. MSP analyses after the treatment (Figure 2a) confirmed that promoter demethylation had occurred in all methylated cell lines by the appearance and enhancement of signals indicative of unmethylated DNA sequences. Those cell lines initially bearing a methylated *DKK3 *promoter showed elevated *DKK3 *mRNA expression after treatment (BT20, MCF7, MDA-MB231, T47D and ZR75-1; Figure 2b), whereas no *DKK3 *mRNA gain was achieved in unmethylated MCF12A and only a marginal *DKK3 *mRNA gain in SKBR3 cells. The induction of *DKK3 *mRNA transcription after the treatment as determined by realtime PCR ranged from 4.7-fold to 29-fold higher than in originally methylated breast cancer cells.

### Differential *DKK3 *mRNA expression in primary breast carcinomas

**Figure 3:**
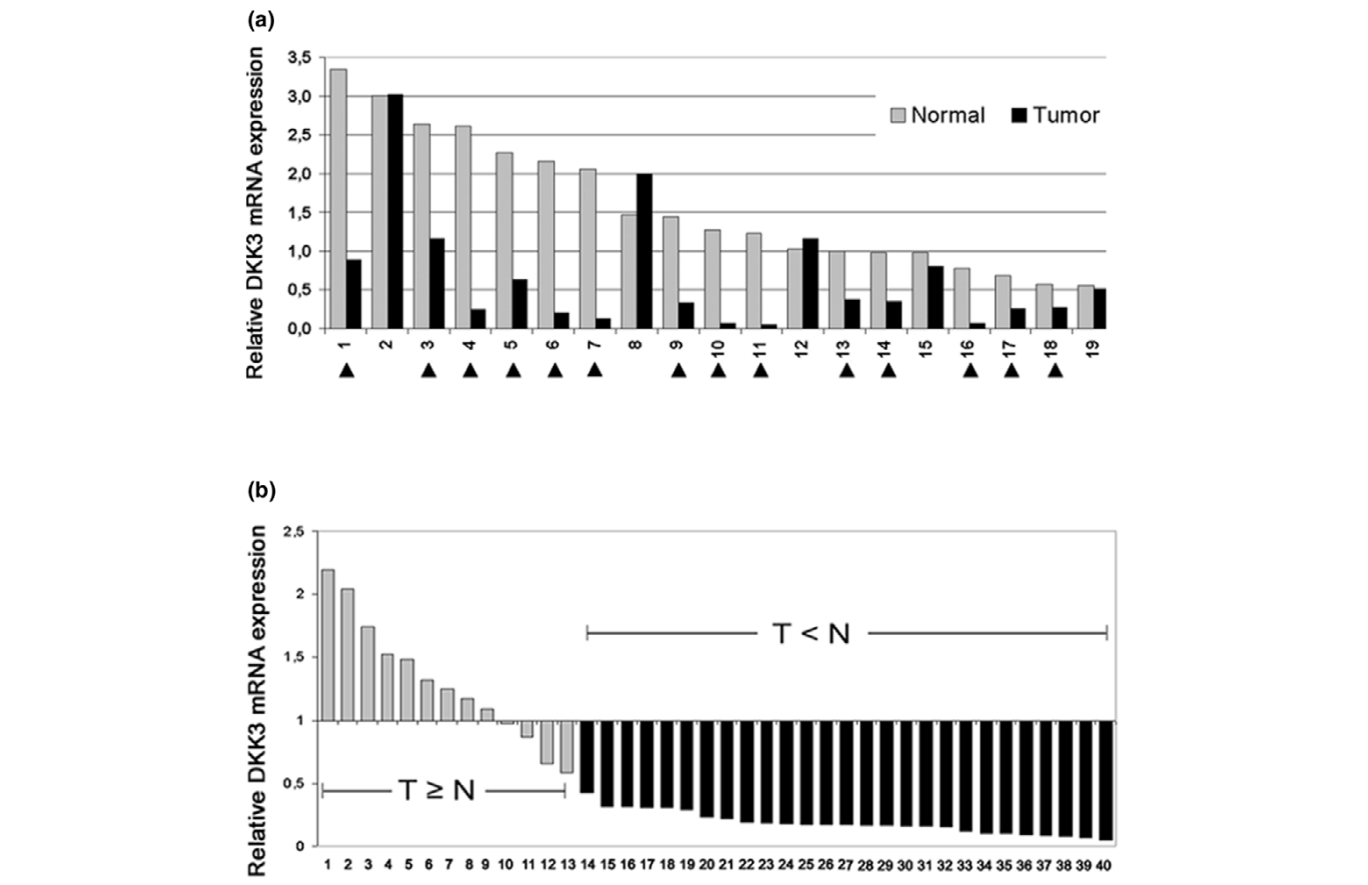
***DKK3 *mRNA expression analysis in primary breast carcinomas**. **(a) **Realtime PCR of *DKK3 *mRNA expression in 19 matched pairs (normal *vs*. tumour). Arrowheads indicate downregulation in the tumour by an expression fold change of more than two (FC2). **(b) **Relative *DKK3 *mRNA expression in 40 additional breast cancer specimens. Mean expression of 19 normal breast tissues (N) was set to 1. Based on a FC2, grey bars represent tumour specimens (T) showing normal expression, black bars represent tumour specimens with reduced *DKK3 *mRNA expression.

*DKK3 *mRNA expression in primary breast tissues was then examined by realtime PCR. In the first step, 19 pairs of breast carcinoma tissues and corresponding normal breast tissue were analysed. A significant downregulation of *DKK3 *mRNA expression in tumour tissue compared with its adjacent normal tissue was detected in 14 (74%) of the 19 pairs (Figure 3a), as defined by an expression fold change of two or more (FC2).

To further support this data set we analysed 40 additional breast carcinomas with no corresponding normal breast tissue and referred each individual *DKK3 *mRNA expression level to the mean *DKK3 *expression of the previously analysed 19 normal breast tissues (Figure 3b). The frequency of *DKK3 *mRNA downregulation measured in this tumour cohort (27 of 40, 68% by FC2) is in good agreement to the result achieved with the 19 matched pairs. Therefore downregulation of *DKK3 *mRNA in breast cancer is considered to affect about 70% of patients.

### *DKK3 *promoter methylation in primary breast carcinomas

**Figure 4:**
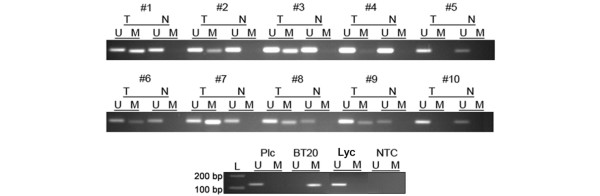
***DKK3 *promoter methylation analysis in primary breast carcinomas**. Methylation specific PCR results from 10 representative matched pairs of primary breast tumour (T) and corresponding normal breast tissue (N) are shown. DNA from human placenta (Plc) as well as from peripheral blood lymphocytes (Lyc) reveals an unmethylated *DKK3 *promoter. DNA from breast carcinoma cell line BT20 served as positive control. NTC = no template control.

To address the question of whether *DKK3 *promoter methylation occurs in primary breast carcinomas, we analysed 150 mammary tumour samples by MSP. Corresponding normal breast tissue was available for 19 tumours. In total, 92 of 150 tumours (61.3%) revealed *DKK3 *promoter methylation (for example, #7 in Figure 4) whereas in 58 of 150 tumours (38.7%) the *DKK3 *promoter was unmethylated. In these cases, MSP amplification signals were obtained exclusively in the U-reaction (for example, #5 in Figure 4). Of the normal breast tissues, only a single sample (5.3% of 19 samples) gave a very weak methylation signal (data not shown) in contrast to all other normal tissues that lacked *DKK3 *promoter methylation. As an additional control that infiltrating immune cells do not contribute to methylation signals in mammary tumours, bisulphite-converted DNA from human peripheral blood lymphocytes were assayed and revealed an unmethylated *DKK3 *promoter, consistent with results from a previous study [39].

### Correlation of *DKK3 *promoter methylation with loss of *DKK3 *mRNA expression

**Figure 5:**
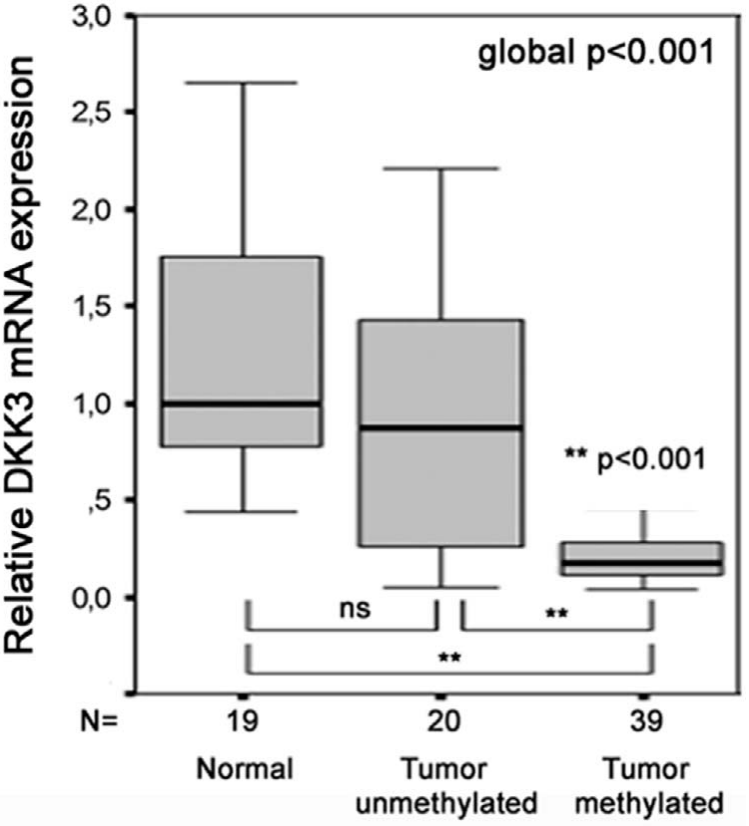
**Correlation analysis of *DKK3 *promoter methylation with *DKK3 *mRNA expression in primary breast carcinomas**. Tumours that are unmethylated in the *DKK*3 promoter express *DKK3 *mRNA comparable with *DKK3 *mRNA expression normal breast tissue, whereas in *DKK3 *methylated tumours *DKK3 *mRNA expression is significantly reduced. Horizontal lines = group medians; boxes = 25 to 75% quartiles, range, peak and minimum.

For all breast carcinoma samples analysed for *DKK3 *mRNA expression (n = 59) we determined the *DKK3 *promoter methylation status at the same time. Thus, we were able to directly compare *DKK3 *methylation and mRNA expression in primary human breast carcinomas. A boxplot (Figure 5) illustrates the distribution and medians of *DKK3 *RNA expression among normal breast tissues, *DKK3 *unmethylated tumours and *DKK3 *methylated tumours. The median *DKK3 *expression level (exp) of unmethylated tumours (exp = 0.87; FC = 1.2) was comparable with that of normal breast tissues (set to exp = 1). In contrast, *DKK3 *methylated tumours showed a significant mRNA downregulation (exp = 0.17; FC = 5.9) compared with *DKK3 *unmethylated tumours and normal breast tissue (global p < 0.001).

### Differential *DKK3 *protein expression in primary breast carcinomas

**Figure 6:**
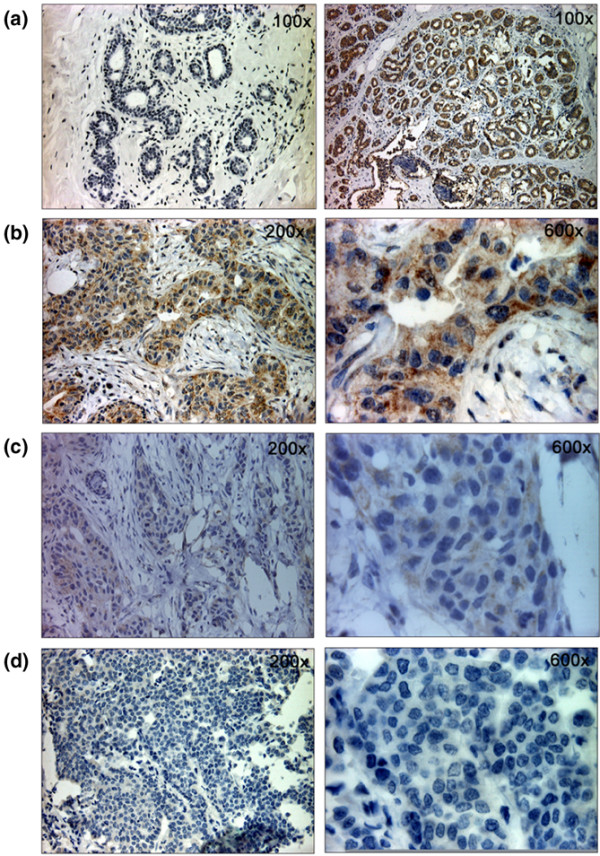
***DKK3 *protein expression in primary breast carcinomas as determined by immunohistochemical staining**. **(a) **Normal mammary tissue without (left) and with application (right) of *DKK3 *antibody. Abundant *DKK3 *protein expression is detectable in luminal and basal epithelial cells. **(b) **Breast carcinoma with unmethylated *DKK3 *promoter reveals abundant *DKK3 *protein expression. **(c) **and **(d) **Breast carcinomas with a methylated *DKK3 *promoter exhibit substantial loss of *DKK3 *protein expression. Original magnifications are given in upper right-hand corner.

Immunohistochemical staining was used to investigate DKK3 protein expression in normal (n = 8) and malignant (n = 16) breast tissues. DKK3 was strongly expressed in non-malignant luminal and basal epithelial cells, achieving a mean (sd) immunoreactivity score (IRS) [34] of 11.3 (1.4) (Figure 6a) and a median IRS of 12 (range 9 to 12). DKK3 protein was not detectable in stromal cells in the normal breast tissue. Of the breast carcinomas, four of 16 (25%) revealed abundant DKK3 protein expression (IRS = 9 to 12; Figure 6b) in contrast to seven of 16 tumours (44%), which showed partial loss (IRS = 4 to 8; Figure 6c), and five of 16 tumours (31%) with substantial loss of DKK3 protein (IRS = 0 to 3; Figure 6d and Table 3). The mean protein staining intensity in breast carcinomas was determined to have an IRS of 5.9 (3.3) and the median to have an IRS of 6 (range 0 to 12). DKK3 expression levels in the tumour and normal tissue groups were shown to be significantly different (p = 0.002; U-test), and DKK3 protein was also differentially expressed within the eight matched pairs (p < 0.001; t-test). As a continuous variable, a lower IRS in breast carcinoma was significantly associated with the presence of *DKK3 *promoter methylation (p = 0.001; Fisher's exact test).

### Association of *DKK3 *promoter methylation with clinicopathological factors

For descriptive data analysis clinicopathological patient characteristics were correlated with the *DKK3 *promoter methylation status. In a bivariate analysis, *DKK3 *promoter methylation was significantly associated with advanced patient age at diagnosis (p = 0.007). Furthermore, *DKK3 *promoter methylation was not associated with tumour size, lymph node status, histological grade, histological type, and oestrogen receptor or progesterone receptor positivity (data not shown).

## Discussion

It was previously reported that expression of the putative Wnt antagonist *DKK3 *was downregulated in several tumour entities as a consequence of epigenetic DNA modification [15,16,18,20-22,24]. Our study is the first to analyse *DKK3 *gene regulation in human breast cancer. Malignant breast cell lines showed strong reduction of *DKK3 *mRNA in association with *DKK3 *promoter methylation. Consistently, *DKK3 *mRNA expression was induced after promoter DNA demethylation in these cells. In primary breast carcinomas, *DKK3 *mRNA expression was downregulated in 68% of invasive tumours with significant association with methylation of the *DKK3 *gene promoter (p < 0.001). The total frequency of *DKK3 *methylation was 61% in breast carcinomas, whereas corresponding normal breast tissues were unaffected by this epimutation. We further showed that a loss of DKK3 protein in breast carcinomas is also associated with *DKK3 *promoter methylation (p = 0.001) whereas protein expression is abundant in epithelial cells of the normal breast. In summary, our data demonstrate for the first time that promoter methylation-mediated downregulation of *DKK3 *expression is a frequent and tumour-related epigenetic alteration in the development of human breast cancer.

The implication of aberrant canonical Wnt/β-catenin signalling in the pathogenesis of human cancer has become widely accepted [40]. Its oncogenic effect is mediated by uncontrolled activation of target genes that for example, enhance cell proliferation, such as c-myc and cyclin D1. In breast cancer, several genes encoding inhibitors of canonical Wnt/β-catenin signalling have been reported to be frequently hypermethylated, for example, *SFRP1 *[34,41], *SFRP2 *[42], *SFRP5 *[43], *WIF1 *[44] and *DKK1 *[42]. We suggest that disruption of a non-canonical Wnt signalling branch, the PCP pathway, may also be implicated in human carcinogenesis by pathologically altering the networks of cellular adhesion, motility and cell polarity, because it has been shown that expression of the putative PCP pathway inhibitor *DKK3 *is commonly downregulated in malignant tissues. As a consequence, loss of *DKK3 *may promote hyperactivation of the PCP pathway, thereby potentially enhancing tumour aggressiveness.

Recent *in vivo *experiments support a hypothesis that the loss of *DKK3 *expression promotes an aggressive cancer phenotype. In a mouse model, *DKK3 *proved to be a promising therapeutic agent to significantly inhibit tumour growth in testicular germ cell cancer [14]. In an orthotopic prostate cancer model a similar treatment resulted in tumour regression, decreased metastasis and prolonged survival of the host [13]. The most recent findings from a breast cancer study revealed that *DKK3 *not only attenuates tumour growth in a xenotransplantation mouse model, it also re-sensitised multidrug-resistant MCF7/ADR cells to doxorubicin treatment in a JNK-c-Jun dependent manner [26]. This highlights its potential utility as a gene therapeutic agent in human breast cancer. Our study adds important information to this aspect, because it so far remained unknown if methylation-mediated loss of *DKK3 *expression also occurred in primary breast cancer, and, if so, how many patients were affected by this epimutation. We have shown that a large proportion (61%) of breast cancer patients have *DKK3 *promoter methylation in the carcinoma tissue, leading to a functional inactivation of the tumour-protective protein. Therefore, we conclude that a potential gene therapeutic treatment with *DKK3 *might be of benefit for a large target population of breast cancer patients.

In contrast to other studies, *DKK3 *promoter methylation in our cohort was not associated with clinicopathological factors indicative of a progressive cancer subtype, such as tumour size, node status or histological grade. The existence of such an association has been demonstrated in prostate cancer [7,12], in which expression of *DKK3 *was predominantly lost in high-grade prostatic tumours. Moreover, siRNA-mediated downregulation of *DKK3 *expression in prostate epithelial cells disrupted acinar morphogenesis [7], which taken with its prevalent expression in growth-arrested cells suggests a functional role of *DKK3 *in post-mitotic tissue differentiation processes. Whether *DKK3 *is also involved in maintaining glandular morphology in the normal mammary gland will be elucidated in a further study.

In human breast cancer, hypermethylation of Wnt antagonist genes was reported to be of clinical relevance. Both *SFRP1 *and *SFRP5 *methylation were shown to occur frequently and be tumour specific with a strong association to poor clinical patient outcome [34,43]. An impact of *DKK3 *promoter methylation on cancer patient survival has been repeatedly found. It was shown to be associated with reduced disease-free survival in acute lymphoblastic leukaemia [20], and also with shorter overall survival in kidney cancer [45] and lung cancer [46]. Due to its functional properties as a potential tumour suppressor in human cancers including breast cancer [26], together with the finding that *DKK3 *methylation is a significant prognostic factor in three human malignancies, we speculate that *DKK3 *methylation might also bear prognostic power in breast cancer. This hypothesis is currently being approached in our laboratory in a further study.

In summary, we demonstrate for the first time that the Wnt antagonist gene *DKK3 *is a frequent target of epigenetic inactivation in human breast cancer, leading to downregulation of *DKK3 *mRNA and DKK3 protein expression in tumourous tissues. These results suggest a causative implication of *DKK3 *in the development of human breast cancer. Since *DKK3 *is believed to negatively regulate Wnt signalling, these results underline the pivotal role of a deregulated Wnt signalling pathway commonly found in this disease.

## Conclusion

This study shows that the putative Wnt antagonist *DKK3 *is frequently downregulated in human breast cancer due to promoter methylation, whereas it is abundantly expressed and unmethylated in normal breast cell epithelium. Since promoter methylation is a primary cause to functionally inactivate tumour suppressor genes, *DKK3 *may act as a tumour suppressor in the human mammary gland. *DKK3 *is believed to particularly regulate non-canonical Wnt signalling. Therefore, we conclude that disruption of this Wnt pathway branch may add further tumour growth advantages to those already conferred by canonical Wnt/β-catenin signalling.

## Abbreviations

DKK3: Dickkopf-3; FC: fold change; IRS: immunoreactivity score; JNK: c-Jun-kinase; LRP: LDL-receptor related protein; MSP: methylation-specific polymerase chain reaction; PCP: planar cell polarity pathway; RT-PCR: reverse transcription polymerase chain reaction; SD: standard deviation; siRNA: small interfering ribonucleic acid; TSA: trichostatin A.

## Competing interests

Edgar Dahl has declared that he has submitted a patent application on the use of *DKK3 *promoter methylation. The other authors have no competing interests.

## Authors' contributions

JV carried out the gene expression analyses, immunohistochemical studies, methylation experiments and statistical evaluations, participated in the conception and design of the study, and wrote the manuscript. NB participated in the immunohistochemical analysis, performed data interpretation and critically revised the manuscript. AH provided clinical samples and clinicopathological data, performed data interpretation, supported in statistical analyses and critically revised the manuscript. GK provided clinical samples and clinicopathological data, performed data interpretation and critically revised the manuscript. UH provided clinical samples and clinicopathological data, participated in data interpretation and critically revised the manuscript. RK participated in the design and co-ordination of the study and critically revised the manuscript. ED planned and co-ordinated the study, and critically revised the manuscript. All authors have given final approval of the text to be published.
